# Advances in Procedural Echocardiographic Imaging in Transcatheter Edge-to-Edge Repair for Mitral Regurgitation

**DOI:** 10.3389/fcvm.2022.864341

**Published:** 2022-03-28

**Authors:** Yiting Fan, Jeffrey Shi Kai Chan, Alex Pui-Wai Lee

**Affiliations:** ^1^Department of Cardiology, Shanghai Chest Hospital, Shanghai Jiao Tong University, Shanghai, China; ^2^Laboratory of Cardiac Imaging and 3D Printing, Li Ka Shing Institute of Health Science, The Chinese University of Hong Kong, Hong Kong, China

**Keywords:** echocardiography, 3D TEE, TEER, mitral regurgitation, transillumination imaging

## Abstract

Transcatheter edge-to-edge repair (TEER) therapy is recommended by the American College of Cardiology/American Heart Association (ACC/AHA) guidelines for selected patients with symptomatic severe or moderate-severe mitral regurgitation (MR). Echocardiography, in particular transesophageal echocardiography (TEE), plays a critical role in procedural planning and guidance for TEER. Recent innovations and advances in TEE techniques including three-dimensional (3D) imaging, unlimited x-plane imaging, live 3D multiplanar reconstruction, as well as transillumination imaging with color Doppler and transparency rendering have further enhanced procedural imaging for TEER, especially for complex diseases including commissural defects, clefts, and multi-segment pathologies. This review discusses the technology of these advanced procedural imaging techniques and provides a “step-by-step” guide on how to apply them during the TEER procedure with a focus on their added values in treatment of complex valve lesions.

## Background

Transcatheter mitral valve (MV) repair is a minimally invasive technique for treatment of selected patients with moderate-severe or severe (3+ or 4+) mitral regurgitation (MR). While several technologies are in clinical development, a transcatheter edge-to-edge repair (TEER) device, the MitraClip™ (Abbott Vascular, Santa Clara, CA, US), is currently the only US Food and Drug Administration approved device for transcatheter MV repair (1–3). Other investigational TEER devices include the PASCAL™ (4) (Edwards Lifesciences, Irvine, CA, US) and the DragonFly™ (5) (Valgen Medical, Hangzhou, China) transcatheter MV repair systems. TEER therapy is recommended by the 2020 ACC/AHA guidelines for selected patients with primary and secondary MR (6). Based on the surgical Alfieri edge-to-edge repair, the MitraClip™ system utilizes a cobalt chromium clip covered with a polypropylene fabric that grasps both the anterior and posterior MV leaflets, thereby reducing MR by increasing the coaptation between the regurgitant valve leaflets. According to the data from the manufacturer, over 100,000 patients have been treated with the MitraClip™ worldwide over the past 17 years. Early experience with the use of TEER therapy was confined to patients with favorable anatomy for the repair procedure, namely, A2-P2 defect, limited flail gap (<10 mm) and width (<15 mm), larger MV area (>4 cm^2^), long mobile leaflet (≥7 mm), without commissural lesions or clefts (2, 3). With growing implantation experience, technological improvement in device design, and cumulating outcome data demonstrating clinical benefits, TEER therapy is increasingly used in patients with more complex lesions and less favorable anatomy, including commissural prolapse, multiple lesions, and clefts. Procedural steps common to all TEER systems include analysis of MV anatomy and function, transseptal access to the left atrium (LA), steering of the clip toward MV, and grasping of MV leaflets; these steps are performed under careful procedural imaging guidance, with transesophageal echocardiography (TEE) as the primary imaging modality, and fluoroscopy as adjunct technique. Two-dimensional (2D) TEE has been the standard procedural imaging technique during early clinical experience of the MitraClip™ procedure; the advent of three-dimensional (3D) TEE technology has further enhanced procedural guidance. A combination of 2D and 3D TEE is increasingly used (7–9). The widened spectrum of MV lesions with increased complexity that can now be treated with TEER therapy has stimulated a rapid parallel advances of ultrasound hardware and software to match the increasingly sophisticated procedural imaging demand. In this review, we discuss the technological advances in procedural TEE imaging, and provide a step-by-step guide to applying these new techniques in the TEER procedure.

## Advanced Techniques for Teer Procedural Imaging—Technological Considerations

Introduction of the 3D fully sampled matrix array TEE transducer in the past decade has made possible simultaneous multiplane (or x-plane) and live 3D imaging; more recently, increase in the computing power of ultrasound system and innovations in 3D rendering techniques have led to advances in new imaging techniques. Three new techniques relevant to TEER procedural imaging will be discussed—namely, x-plane imaging with unlimited plane combination, live 3D multiplanar reconstruction (MPR), and transillumination imaging (TI).

## X-Plane Imaging With Unlimited Plane Combination

X-plane imaging displays two views from the same heartbeat utilizing one acoustic window. The default images are orthogonal (90°) to each other. Typically, the left sector displays the reference imaging plane (A plane) and the right sector displays an adjustable plane (B plane) on which the elevation, lateral tilt, and rotation can be manipulated. X-plane imaging allows visualization of anatomic structures and devices from different imaging planes simultaneously, leading to improved accuracy of measurement and device positioning. In the earlier versions of x-plane imaging offered by most vendors, however, there was a limited combination of plane tilting and rotation—the scan planes are forced to align orthogonal when B plane is tilted and tilting always resets when B plane is rotated (10). This limitation becomes important when commissural MR is treated, as the proper clip arm orientation is rotated to maintain perpendicularity to the line of coaptation at the commissures, where the bicommissural view and the optimal leaflet grasping views are non-orthogonal. In the recently updated version of x-plane imaging, combination of plane tilting and rotation is unlimited, allowing flexible multiplane evaluation with scan planes aligned to cardiac structures with non-orthogonal views when required (11) (Supplementary Figure 1).

## Live 3D MPR

The 3D data set can be sliced to generate multiple 2D views (as orthogonal planes, parallel slices, or rotated around a common rotation axis)—a process known as MPR (9). MPR allows 2D visualization of cardiac structures which are difficult (sometimes impossible) to obtain directly from traditional 2D imaging. Conventional MPR is performed offline after the 3D data sets are acquired and stored. Live 3D MPR is a new 3D visualization tool that allows free MPR manipulation during real-time imaging for structure/device alignment and measurements (11, 12). Use of live 3D MPR allows simultaneous display of multiple imaging planes (typically two long-axis views and one short-axis view), as well as a 3D en face view, without or with color Doppler. MPR orientation is typically displayed on the volume data as reference view lines. There are several advantages for using live 3D MPR in the guidance of the TEER procedure: (1) the errors of parallax (perceived shift in position of a structure when it is viewed from different angles on 3D echocardiography) can be minimized by simultaneously displaying the 2D views and 3D images; (2) 2D views that are physically impossible to obtain with conventional 2D imaging as a result of the limitation of physical angle of insonation can be reconstructed and displayed; and (3) the need to change views and probe positions for complete assessment can be avoided.

## Photorealistic Transillumination Imaging and Transparency Rendering

Optimal methods for displaying 3D images are critical for the perception of depth and to provide a clinically useful, high-fidelity images of cardiac structures and devices. Conventional 3D rendering uses shading techniques to encode voxels based on their distance, gray-level gradient, and texture to generate a 3D display of cardiac structures; these techniques do not consistently provide images with adequate detail definition and depth perception. A novel rendering technique known as transillumination imaging (TI) adds a virtual light source and simulates light-tissue interactions, including absorption, scattering and reflection, resulting in a photorealistic image with shadows that highlight structures and enhance depth perception (13, 14). The light source is freely movable within the volume to illuminate specific structures. It has been shown that TI enhances the sense of depth and space, producing images that appear more realistic to the human eye, which facilitates the detection of subtle structures and pathologies, such as ruptured chordae and clefts. TI with transparency rendering is a modification of the TI technique that highlights interface between tissue and blood within the 3D data set (15). TI with transparency displays tissue as transparent while showing the blood/tissue border as a colored contour, allowing the operator to adjust the degree of transparency in order to provide better delineation of cardiac anatomy and devices. Additionally, TI has the ability to superimpose 3D color Doppler onto 3D anatomic data with tissue transparency in order to refine the visualization of MR jet origin before and after the TEER procedure (16).

## Procedural Imaging in Teer Using Advanced Echocardiographic Techniques—Step-by-Step

Procedural imaging in TEER should follow a standard protocol involving the following steps: preprocedural analysis of the MV anatomy and function, transseptal puncture, introduction of the steerable guide into the LA, steering and positioning of the clip, grasping, leaflet insertion assessment, and pre-deployment evaluation. An integrative imaging approach combining the proper use of 2D, Doppler, and advanced 3D techniques as indicated by the imaging goal of each procedural step should be adopted. This review highlights the steps in which the abovementioned advanced imaging techniques have added value, especially for complex lesions, and describes the practical steps to apply them during the procedure.

## Pre-Procedural Analysis of Valve Anatomy and Function

**Figure 1:**
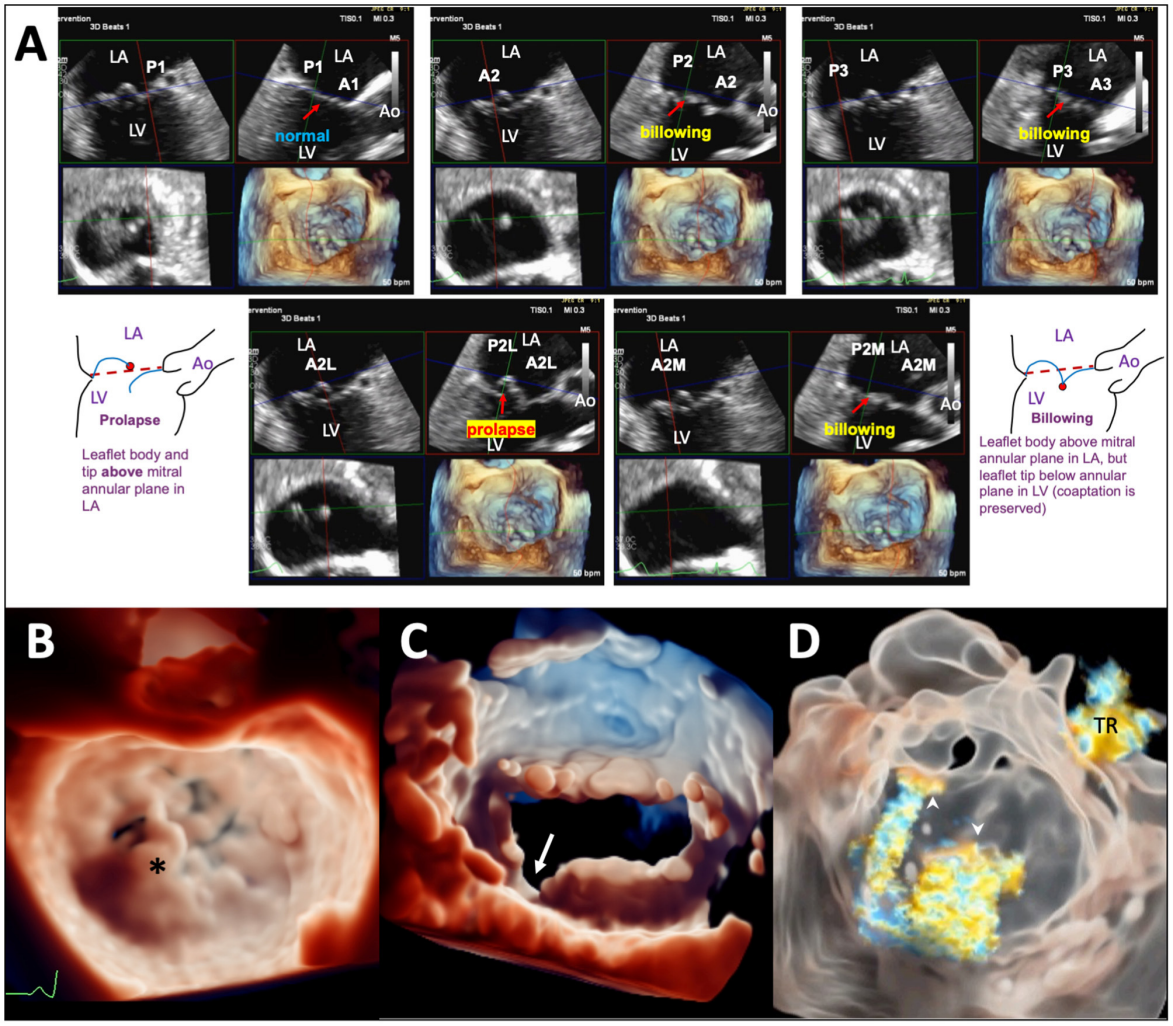
Pre-TEER analysis of MV anatomy and function using advanced TEE imaging techniques. **(A)** Segmental analysis of leaflet edge anatomy using live 3D MPR on 3D TEE data set of a patients with diffuse myxomatous MV disease. Prolapse is seen in the lateral part of the A2 segment (A2L); other segments show either leaflet billowing or normal leaflet motion. **(B)** TI in a patient with a flail P2 segment (*). **(C)** TI of the MV viewed from the LV perspective highlighting a leaflet cleft/deep indentation (arrow) between P2 and P3. **(D)** TI with transparency rendering in a patient with MR of mixed etiologies showing the origins of 2 separate MR jets (arrowheads), one at A2-P2 (secondary MR component) and another from a perforation of the anterior leaflet near the left trigone (primary MR component). A tricuspid regurgitation (TR) jet is also visualized. Ao, aorta; LA, left atrium; LV, left ventricle; A2L, lateral 1/3 of A2; A2M, medial 1/3 of A2; P2L, lateral 1/3 of P2; P2M, medial 1/3 of P2.

Procedural imaging for TEER starts with pre-procedural planning of clips strategy (location, size, and numbers of clip to be implanted) with detail analysis of the valve anatomy and function. The Carpentier nomenclature of the MV scallops divides the valve into 6 segments (A1, A2, A3, P1, P2, and P3). Live 3D MPR is particularly suited for segmental analysis of leaflet edge anatomy. Typically, the reconstructed bicommissural view, long-axis view, short-axis MV view, and a 3D en face image are displayed. By positioning the long-axis plane at each leaflet segment, the leaflet edge anatomy and function of each segment is systematically evaluated along the whole line of coaptation (Figure 1A); any prolapse, flail, billowing, tethering, clefts, and calcifications are documented. In fact, as the lesion location and the optimal clip implantation position can vary by only a few millimeters, a TEER-specific nomenclature dividing the leaflets into more, smaller segments may be required for more precise localization and communication (e.g., the lateral 1/3 of P2 can be called P2L). Segmental analysis with live 3D MPR is particularly important for treating complex lesions with multi-segment pathologies. In degenerative MR due to diffuse myxomatous disease, it is important to differentiate prolapsing from billowing segments, as the former has genuine loss of leaflet edge coaptation (hence becomes the MR origin) and could be the primary target site for clip implantation (1, 17, 18). Pure billowing, on the other hand, does not contribute to MR and may not require clipping. Calcifications of leaflet grasping zone may preclude clip implantation in the relevant segment, and clefts or deep indentations may need specialized clips strategy (19). The added advantage of live 3D MPR over plain 2D (including simultaneous biplane) imaging is the simultaneous display of 2D planes and 3D views, allowing guided alignment of the reconstructed long-axis 2D plane to be perpendicular to the line of coaptation; this technique allows the length of leaflet segment available for grasping to be measured with improved accuracy. Similarly, the flail width, flail gap, and MV area can be measured with higher accuracy by adjusting the 2D planes to the correct positions according to the anatomy (20, 21). Importantly, interrogation of a smaller 3D volume can be performed during 3D imaging to compensate for the relative reduction of spatial resolution and frame rates, reducing measurement error. The 3D image of the MV should be viewed from both the atrial (Figure 1B) and ventricular (Figure 1C) perspectives for visualization of leaflet clefts or indentations, which can be better appreciated with the use of TI (Figures 1B,C). TI with color Doppler and transparency rendering could enhance 3D visualization of the origin(s) of the MR jet(s) (16) (Figure 1D).

## Transseptal Puncture

**Figure 2:**
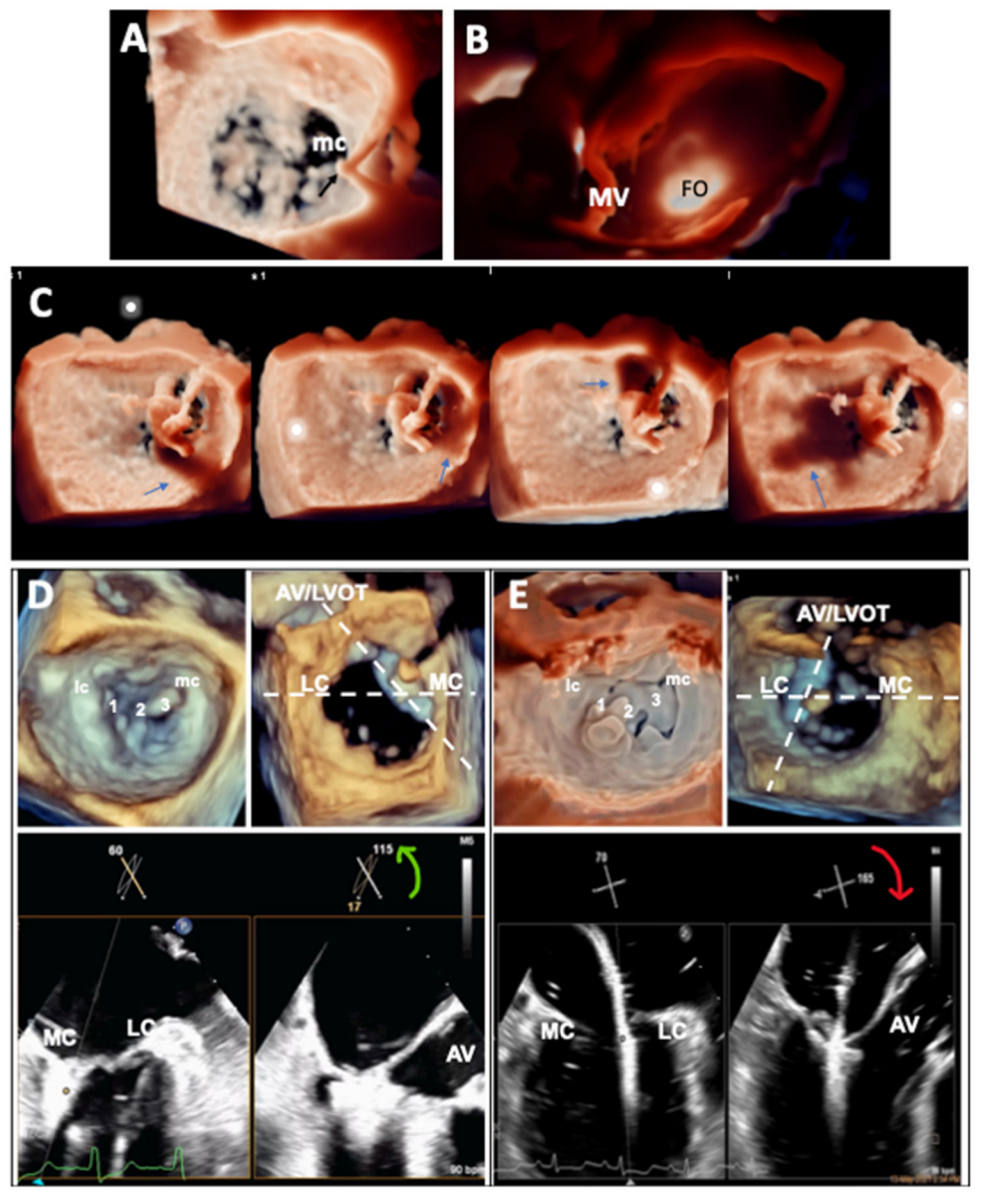
Application of advanced TEE imaging in the guidance of mitral TEER procedure. **(A)** 3D en face view of the MV showing the position of tenting of the atrial septum by the transseptal puncture needle and its relationship with the medial commissure (mc) of the MV. **(B)** TI of the atrial septum showing the position of the fossa ovalis (FO) and its relationship with the MV. **(C)** TI with the virtual light source at different positions in the LA casting shadows (arrows) of the clip to enhance 3D perception of distance of the clip from adjacent structures during clip steering. **(D,E)** X-plane imaging (bicommissural and long-axis views) with simultaneous independent tilting and rotation facilitating leaflet grasping for medial **(D)** and lateral **(E)** commissural prolapse.

The goal of transseptal puncture is to ensure the clip delivery system crosses the atrial septum at an appropriate distance (the transseptal height) from mitral annular level according to leaflet anatomy and function. An optimal transseptal height allows appropriate alignment of the clip to adequate trajectory toward the mitral leaflets for proper grasping. Typically, transseptal puncture is performed at the superior and posterior-mid aspect of the fossa ovalis to achieve a transseptal height of 4–4.5 cm (Supplementary Figure 2). The bicaval (~90°) view and short-axis (~45°) view guide transseptal puncture in, respectively, superoinferior and anteroposterior direction by monitoring tenting of fossa ovalis by the Brockenbrough™ needle. It is important to have the aortic valve visualized in the 45° short-axis view to ensure puncture is performed away from the aorta. However, bi-caval and short-axis planes are in fact ~45° (non-orthogonal) to each other; in the previous version of x-plane imaging, when A plane is used to show the bi-caval view, tilting of B plane to “chase” the needle resets the short-axis B plane to 0°, and aorta becomes out of sight. The new x-plane imaging with unlimited plane combination has important added advantage by allowing tilting of B plane without resetting its rotation to allow visualization of aorta throughout the puncture process and minimizes the chance of aortic injury (Supplementary Figure 3). Fine adjustment of transseptal height (±5 mm) may be required according to leaflet anatomy and function: flail leaflets or prolapse requires a higher while functional MR with tethered leaflets requires a lower transseptal height, owing to the different level of leaflet coaptation relative to the annulus. On the other hand, transseptal height should be higher for medial and lower for lateral commissural MR, because the clip delivery system gains height as it travels laterally. Moreover, the medial commissure is more caudal and the lateral commissure more cephalic; therefore, supero-inferior position of transseptal puncture may need to be adjusted accordingly for treatment of commissural MR (19). Unfortunately, it is difficult to appreciate the relationship between transseptal puncture site and MV commissures on 2D imaging; 3D imaging has added value by demonstrating the distance and position of transseptal puncture relative to mitral commissures and leaflets in the 3D en face view (Figure 2A). Furthermore, TI with lighting source behind fossa ovalis is valuable for assessing soft tissue thickness and enhances 3D perception of anatomic relations with mitral leaflets, providing additional information for guiding transseptal puncture (Figure 2B).

## Clip Steering and Positioning

Once the guide catheter is correctly positioned, the clip delivery system is inserted and advanced through the guide into the LA. As movement of the clip delivery system in LA is 3-dimensional (antero-posterior, septo-lateral, and deflection toward MV), 3D imaging is superior to 2D imaging in guiding the steering process without needing to change views (22). Additionally, TI with lighting source in LA enhances 3D perception by casting shadows of the clip on surrounding structures, and helps to confirm the clip is free from LA wall and valve tissue to avoid cardiac injury (Figure 2C). The goals of steering are to align the clip to a trajectory perpendicular to the plane of the MV and to position the clip to the target lesion/MR origin. X-plane imaging with simultaneous display of the bicommissural and long-axis views, combined with fluoroscopy, is often used to check clip trajectory. However, 2D imaging may occasionally be difficult to confirm axiality of clip trajectory as the clip path may be out of plane despite meticulous probe manipulation. This may be more likely in complex procedures, such as treatment of commissural MR or when the LA is severely dilated. Live 3D MPR has a unique advantage by displaying simultaneously the reconstructed 2D bicommissural plane (M/L alignment) and long-axis plane (A/P alignment), as well as the 3D en face view of the MV (clip arms perpendicularity to line of coaptation); orientations of MPR can be aligned co-axial to the delivery catheter shaft (Supplementary Video 1) and clip trajectory can be observed and adjusted in real-time (23). Failure to confirm that the clip arms are perpendicular to the line of coaptation may result in loss of leaflet capture and insertion. Clip arms perpendicularity is best confirmed on 3D imaging. In the en face “surgeon view”, the open clip arms are oriented 12-6 o'clock (with aortic valve at 12 o'clock) to be perpendicular to the coaptation line at A2/P2; but for peripheral or commissural defects, because the coaptation line is curved like “smiley face” the clip arms should be rotated clockwise or counterclockwise to achieve perpendicularity for, respectively, lateral (A1/P1/lateral commissural) or medial (A3/P3/medial commissural) defects.

## Leaflet Grasping and Pre-Deployment Evaluation

After verification of axial trajectory and clip arms perpendicularity, the clip is advanced into LV; after rechecking clip orientation below the MV, the clip is partially closed and brought up to grasp the leaflets. Leaflet grasping is best performed by using x-plane imaging (bicommissural and long-axis) (24, 25). Proper grasping requires both clip arms to be seen within the long-axis grasping plane. The intersection angles between the bicommissural and grasping planes at the peripheral segments are non-orthogonal (26). For lateral defects (A1/P1/lateral commissure), lateral tilt and clockwise rotation of B plane should get the proper grasping view; for medial defects (A3/P3/medial commissure) medial tilt and counterclockwise rotation will be needed. One easier way to remember which way to rotate is that the B plane rotation follows the same direction as clip rotation to achieve perpendicularity on the 3D *en face* image (Figures 2D,E). Similarly, live 3D MPR can be useful to display the proper bicommissural and grasping plane to monitor leaflet capture in patients whose 2D planes are not easy or possible to obtain with x-plane imaging (27). The orientations of the live-reconstructed 2D planes can be adjusted to follow the device direction and position during real-time grasping (23). Specific structures can be identified and located correctly on the 3D image, which is particularly helpful when multiple clips are implanted (Supplementary Video 2). Leaflet insertion and MR reduction should be confirmed before clip deployment. MPR is helpful in confirming leaflet insertion (23, 28) by calculating the difference of the leaflet length outside the clip measured in the long-axis plane on the clip and the entire leaflet length measured in the parallel planes just beside the clip (Supplementary Figure 4). In addition to the bicommissural/long-axis x-plane imaging, 3D color-Doppler imaging using TI with tissue transparency adds visual information of localization of any residual MR jet origin (16) (Supplementary Video 3).

## Conclusion and Future Perspectives

Advanced procedural TEE 3D imaging and related techniques have incremental practical value in procedural guidance for TEER especially in complex procedures. New hybrid, or fusion, imaging technology allows for 3D TEE to be overlaid on fluoroscopy to provide the most pertinent information from both imaging modalities on a single screen, and may improve safety and efficacy of the TEER procedure (29). In future, technological improvement in 3D imaging especially on resolution and frame rates (30), as well as the advent of full-volume 3D intracardiac echocardiography technique (12), will likely further enhance the use of 3D echocardiography in procedural imaging to simplify and optimize procedural guidance for structural heart interventions.

## Author Contributions

YF and JC were responsible for drafting of the manuscript. AL was responsible for conceptualization and revision of the manuscript. All authors contributed to the article and approved the submitted version.

## Funding

This work was supported by the Hong Kong Health and Medical Research Fund (05160976).

## Conflict of Interest

AL is a speaker and consultant for Abbott Structural and Philips Healthcare. The remaining authors declare that the research was conducted in the absence of any commercial or financial relationships that could be construed as a potential conflict of interest.

## Publisher's Note

All claims expressed in this article are solely those of the authors and do not necessarily represent those of their affiliated organizations, or those of the publisher, the editors and the reviewers. Any product that may be evaluated in this article, or claim that may be made by its manufacturer, is not guaranteed or endorsed by the publisher.
