# Supplementary material for: Advances in Procedural Echocardiographic Imaging in Transcatheter Edge-to-Edge Repair for Mitral Regurgitation
Source: Front Cardiovasc Med. 2022 Mar 28;9:864341. doi: 10.3389/fcvm.2022.864341 (PMC8996060; doi:10.3389/fcvm.2022.864341)
Supplement: Supplementary file 1 [file Table_1.DOCX]

Supplementary Material

## Supplementary Figures

**Figure 1: The old and new x-plane techniques.** (A) Old x-plane imaging of a patient with P3 MV prolapse. The x-plane cursor (arrow) is tilted 14° medially (red box); the right plane, which is orthogonal to the left plane, is not perpendicular to the line of coaptation. (B) New x-plane imaging of another patient with P3 prolapse. The x-plane cursor (arrow) can be tilted (18° medially) and rotated (30° counterclockwise) simultaneously (blue boxes). The right plane can therefore be adjusted to be perpendicular to the line of coaptation. Ao, aortic valve; LA, left atrium; LV, left ventricle.

**Figure 2:** **Transseptal puncture**. The puncture site is evaluated using three transesophageal echocardiography planes: a short-axis view at the base for anterior-posterior orientation (~45°) (blue box), a bi-caval view for superior (cranial)-caudal (inferior) orientation (~90°) (red box), and a standard four-chamber view (~0°) (green box) or a mirror-image four-chamber view (~160°) (purple box). The ‘tenting’ (white arrow) is seen as the trans-septal needle being pushed against the IAS, ideally in the superior and posterior part of the IAS with the aim of obtaining adequate working space and distance above the mitral annulus. Of note, the standard four-chamber TEE view may not be able to visualize tenting at the superior aspect of the fossa ovalis, especially in patients with severe left atrial enlargement. To overcome this limitation, the mirror-image four-chamber view (~160°) can be used, from which the superior aspect of the fossa ovalis can usually be visualized and transseptal puncture height assessed.

**Figure 3:** **X-plane (bicaval and short-axis) imaging with independent tilting and rotation to guide transseptal puncture.** The new x-plane imaging with unlimited plane combination has important added advantage by allowing tilting of B plane without resetting its rotation to allow visualization of aorta throughout the puncture process and minimize the chance of aortic injury.

**Figure 4: Assessment of leaflet insertion using live 3D MPR.** D1 and D3 indicate the residual posterior and anterior leaflet length after clip; D2 and D4 are the length of the leaflets beside the clip. The difference between D1 and D2 is the length of leaflet insertion of the posterior leaflet, and the difference between D3 and D4 is that of the anterior leaflet.

**Supplementary Videos**

**Video 1: Assessment of clip trajectory using live 3D MPR.**

**Video 2: Leaflet grasping by guided by live 3D MPR in a patient requiring multiple clips.**

**Video 3: Assessment of residual MR jet origin after clip implantation using transillumination imaging with tissue transparency rendering.**
