# Supplementary figures and images for: Advances in Procedural Echocardiographic Imaging in Transcatheter Edge-to-Edge Repair for Mitral Regurgitation
Source: Front Cardiovasc Med. 2022 Mar 28;9:864341. doi: 10.3389/fcvm.2022.864341 (PMC8996060; doi:10.3389/fcvm.2022.864341)

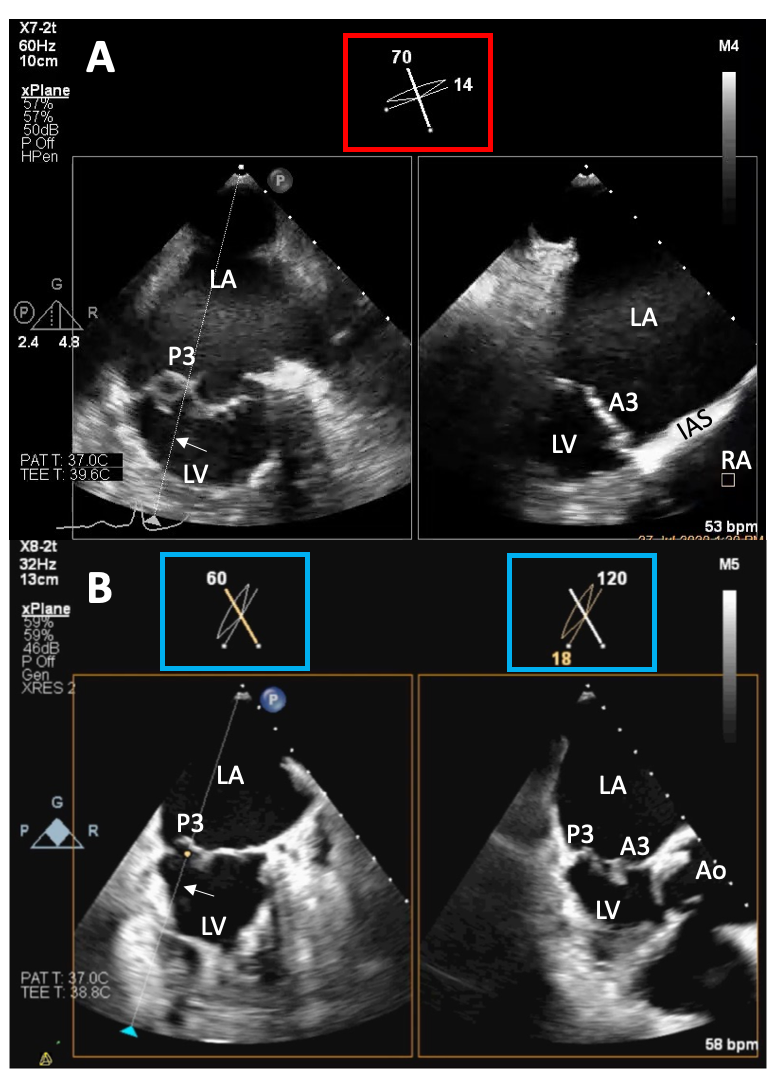

Supplement: Supplementary file 5 [file Image_1.TIFF]

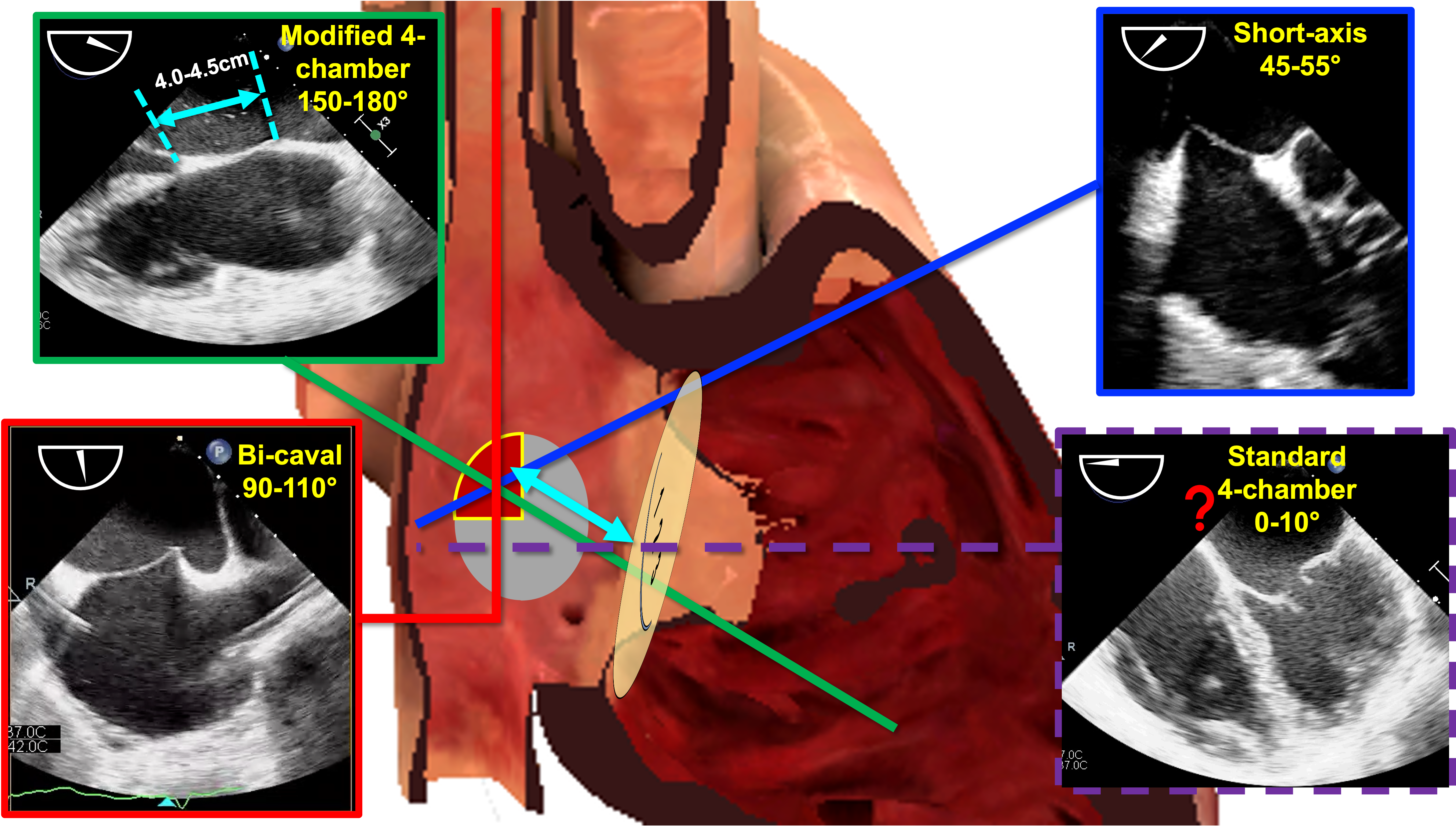

Supplement: Supplementary file 6 [file Image_2.TIFF]

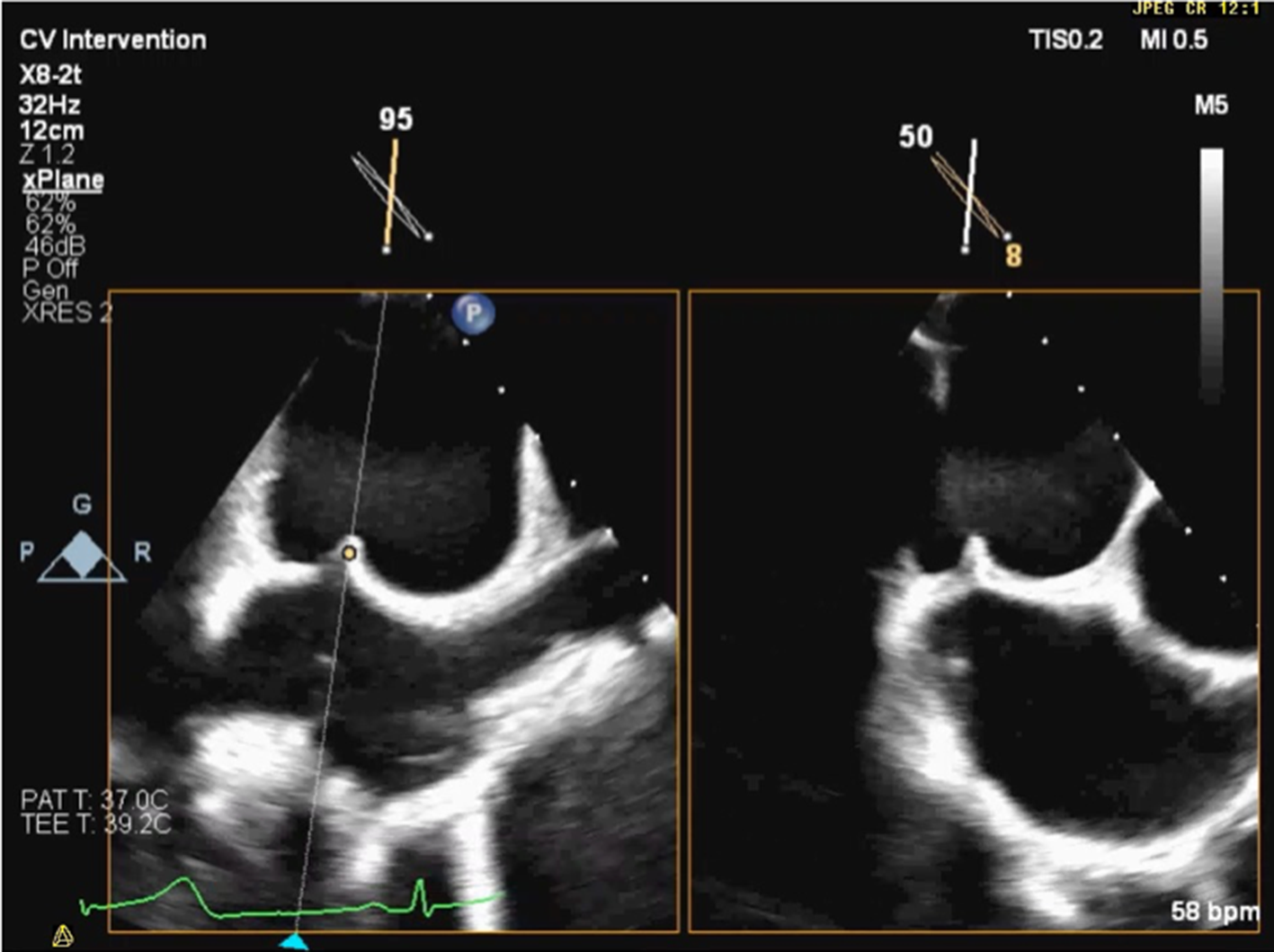

Supplement: Supplementary file 7 [file Image_3.TIFF]

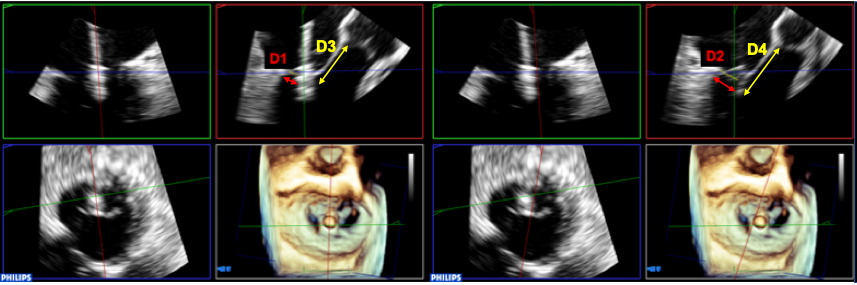

Supplement: Supplementary file 8 [file Image_4.TIFF]
